# Relationship between Birth Weight and Metabolic Status in Obese Adolescents

**DOI:** 10.1155/2013/490923

**Published:** 2013-08-28

**Authors:** David J. Hill, Harry Prapavessis, J. Kevin Shoemaker, Michelle Jackman, Farid H. Mahmud, Cheril Clarson

**Affiliations:** ^1^London Health Sciences Centre, Lawson Health Research Institute, London, ON, Canada N6A 5W9; ^2^Lawson Health Research Institute, St Joseph's Health Care, 268 Grosvenor Street, London, ON, Canada N6A 4V2; ^3^Department of Paediatrics, University of Western Ontario, London, ON, Canada N6A 5A5; ^4^Department of Medicine, University of Western Ontario, London, ON, Canada N6A 5A5; ^5^Department of Physiology and Pharmacology, University of Western Ontario, London, ON, Canada N6A 5A5; ^6^School of Kinesiology, University of Western Ontario, London, ON, Canada N6A 5A5; ^7^London Health Sciences Centre, Children's Hospital, London, ON, Canada N6A 5W9; ^8^Department of Pediatrics , University of Calgary, Calgary, Alberta, Canada; ^9^Department of Paediatrics, University of Toronto and Hospital for Sick Children, Toronto, ON, Canada M5G 1X8

## Abstract

*Objective*. To examine the relationships between birth weight and body mass index, percent body fat, blood lipids, glycemia, insulin resistance, adipokines, blood pressure, and endothelial function in a cohort of obese adolescents. *Design and Methods*. Ninety-five subjects aged 10–16 years (mean age 13.5 years) with a body mass index >95th centile (mean [±SEM] 33.0 ± 0.6) were utilized from two prospective studies for obesity prevention prior to any interventions. The mean term birth weight was 3527 ± 64 g (range 1899–4990 g;). *Results*. Body mass index *z*-score correlated positively with birth weight (*r* ^2^ = 0.05, *P* = 0.03), but not percent body fat. Insulin resistance negatively correlated with birth weight (*r* ^2^ = 0.05, *P* < 0.001), as did fasting plasma insulin (*r* ^2^ = 0.05, *P* < 0.001); both being significantly greater for subjects of small versus large birth weight (Δ Homeostasis Model Assessment = 2.5 and Δ insulin = 10 pmol/L for birth weight <2.5 kg versus >4.5 kg). Adiponectin, but not leptin, blood pressure *z*-scores or peripheral arterial tomography values positively correlated with birth weight (*r* ^2^ = 0.07, *P* = 0.008). *Conclusions*. Excess body mass index in obese adolescents was positively related to birth weight. Birth weight was not associated with cardiovascular risk factors but represented a significant determinant of insulin resistance.

## 1. Introduction

Childhood obesity in Canada, as in other developed countries, has increased dramatically with 29% of children being overweight or obese in 2007 compared to 15% in 1978 [1]. Obesity in childhood is a strong indicator of future obesity in adulthood and also confers a high risk for future development of type 2 diabetes (T2D) and cardiovascular disease [2, 3]. The future risk of adult T2D is greater in adolescents who demonstrate insulin resistance [4]. Small-for-gestational age (SGA, <10th percentile birth weight) and large-for-gestational age (LGA, >90% percentile) birth weight can both confer an increased risk of adolescent and adult obesity, insulin resistance, metabolic syndrome, and T2D in adulthood [5, 6]. A meta-analysis of 20 studies showed that birth weight greater than 4 kg increased the risk of obesity (odds ratio 2.1), but being SGA did not, although the majority of the studies analyzed considered children prior to adolescence [7]. However, it is not clear whether consideration of birth weight is of practical relevance in the management of the individual obese child at risk of the development of metabolic or cardiovascular disease.

The association between LGA, often with macrosomia, and adolescent and adult obesity, as well as the associated increased risk of T2D and cardiovascular disease, is most apparent when accompanied by maternal diabetes, both gestational and pregestational [8, 9]. Maternal pregestational obesity, with or without excessive weight gain during pregnancy, provides an equivalent risk for maternal hyperglycemia and infant macrosomia as does diabetes, and the offspring in both conditions exhibit a greater risk of obesity during adolescence [10, 11]. In pregnancies uncomplicated by diabetes or biased by maternal obesity, increasing birth weight correlated positively with child and adult BMI [12]. However, BMI does not distinguish between lean and fat body mass, or body fat distribution, and birth weight also showed a positive correlation with lean body mass in children and adults [13]. An increase in birth weight equivalent to one standard deviation was associated with an additional kg of body weight during adolescence, but this increase was in fat-free body weight [13]. Although LGA outside of maternal diabetes or obesity may result in a high BMI during adolescence, this has not been shown to be associated with truncal obesity or insulin resistance, or with a greater risk of future metabolic disease [14].

In comparison with LGA, SGA infants have been shown to be at greater risk of hypertension and cardiovascular disease as adults [15]. While SGA was associated with inadequate maternal nutrition, there is evidence of an altered mass of pancreatic insulin-producing *β* cells, insulin release, and insulin resistance from animal models and of glucose intolerance and insulin resistance in the human offspring resulting in an increased risk of diabetes in the adult [16, 17]. In the developed world most SGA infants do not result from nutritionally inadequate pregnancies but can still exhibit abnormal growth trajectories and demonstrate metabolic dysfunction postnatally. SGA infants have a greater risk of childhood obesity, although this is dependent on the trajectory of postnatal catch-up growth. Most SGA infants exhibit some catch-up growth between 6 and 12 months of age [18]. However, those exhibiting rapid catch-up growth prior to 4 years were at greater risk of being obese as adolescents and adults, with a subsequent increased risk of metabolic syndrome and diabetes [19]. The SGA infant who undergoes rapid catch-up growth is more likely to exhibit truncal fat obesity, independent of an increased BMI [20].

Park [21] showed a negative correlation between birth weight and insulin resistance in 34 overweight children, but the relationship between birth weight and metabolic changes in obese adolescents that may predispose to diabetes or cardiovascular disease in adulthood has not been examined in detail. Therefore, we examined the relationships between birth weight and BMI, body fat content, glycemic control, insulin resistance, circulating adipokines, blood lipids, blood pressure, and endothelial function in obese adolescents at baseline as part of obesity prevention programs.

## 2. Methods

### 2.1. Study Design

 Entry data were utilized from 2 prospective cohort studies of obese adolescents. Twenty-six subjects were recruited for an obesity intervention which was completed in 2007, and 69 subjects were screened between 2008 and 2010 for entry to the REACH trial. Both groups were evaluated by the same clinical team and lead physician Cheril Clarson as the initial obesity prevention cohort was used as a pilot group to assess interest and feasibility for a more intensive clinical study. The REACH study (ClinicalTrials.gov number NCT00934570) will assess the effects of a structured lifestyle intervention and metformin (or placebo) on BMI and other risk factors for T2D and cardiovascular disease in obese adolescents. Both studies were approved by the Western University Research Ethics Board, London, ON, Canada, in addition to Health Canada. Informed consent was obtained from all study subjects. 

### 2.2. Subjects

 The study cohorts comprised obese subjects aged 10 to 16 years, defined as BMI greater than the 95th percentile for age and gender. Exclusion criteria included a fasting plasma glucose ≥6.0 mmol/L and within the REACH study a 2 h glucose value ≥11.1 mmol/L following an oral glucose tolerance test and a glycosylated hemoglobin (A1C) of ≥6% (≥7 mmol/L). Pubertal staging was assessed using the method of Tanner. At study entry, potential risk factors for development of T2D in addition to obesity were assessed, including pregestational maternal type 1 or 2 diabetes or gestational diabetes, birth weight, ethnicity, and the presence of acanthosis nigricans, hirsutism, or acne in adolescent girls.

### 2.3. Measurements

 All subjects were asked to fast from 10 p.m. of the evening prior to study and to avoid caffeine for the preceding 24 h period. Blood was sampled for measurement of glucose, insulin, LDL and HDL cholesterol, triglycerides, and the adipocytokines, leptin, and high molecular weight adiponectin. Subjects (69) entering the REACH study also had an oral glucose tolerance test (2 h GTT) with measurement of 2 h plasma glucose and A1C. Glucose was measured by the glucose oxidase technique and plasma insulin levels determined by a double antibody radioimmunoassay using a recombinant human insulin standard and the minimum detection level of <2 pmol/L. Within- and between-assay coefficients of variation were 5.8% and 6.5%, respectively. Insulin resistance was estimated by the measurement of Homeostasis Model Assessment (HOMA), calculated as fasting plasma insulin (mU/L) × fasting plasma glucose (mmol/L)/22.5. HOMA values greater than 3.0 in adolescents are indicative of insulin resistance [22]. Serum leptin levels were measured using an ELISA assay (Quantikine, R&D Systems Inc., Minneapolis, MN) with a sensitivity of less than 8 pg/mL and inter- and intra-assay coefficients of variation of 5% and 3%, respectively. High molecular weight adiponectin was also measured with an ELISA (ALPCO Diagnostics, Salem, NH) with a sensitivity of less than 19 pg/mL and inter- and intra-assay coefficients of variation of 6% and 3%, respectively. A1C was measured by radioimmunoassay by clinical laboratory services, London Health Sciences Centre, London, ON, Canada.

Weight was measured with subjects wearing minimal clothing using an electronic scale to the nearest 0.1 kg. Height was measured without shoes using a wall mounted stadiometer to the nearest 0.1 cm. BMI *z-*scores were calculated from the US Centers for Disease Control and Prevention reference data [23]. The percent body fat was determined by whole body dual-energy x-ray absorptiometry (DEXA) analysis (General Electric-Lunar iDXA, Mississauga, ON, Canada).

Diastolic and systolic blood pressure was measured in the right arm using an appropriately sized cuff using a Dynamap machine. Age and sex-adjusted *z-*scores were calculated using the National High Blood Pressure Education Program Working Group on High Blood Pressure in Children and Adolescents [24]. A measurement of endothelial function was obtained using peripheral arterial tonometry (PAT) with lower RH-PAT values indicative of greater endothelial dysfunction (Itamar Medical, Israel). Details of this technique have been described previously [25].

### 2.4. Statistical Analysis

Measured values were obtained from 95 individuals, except for values for A1C, 2 h GTT, and percent body fat, which were obtained from a subgroup of 69 subjects. The normality of variables was confirmed using the Kolgomorov-Smirnov test. Values are expressed as mean ± SEM. The relationships between BMI and diabetes and cardiovascular risk factor variables at study entry were examined by univariate linear regression, as were the associations between birth weight and the following factors: BMI *z-*score, percent body fat, blood pressure *z-*scores, plasma insulin, leptin, adiponectin (and leptin/adiponectin ratio), HOMA, fasting and 2 h GTT plasma glucose, circulating lipids (HDL and LDL cholesterol and triglycerides), A1C, and RH-PAT. Stepwise regression with forward entry was also used to investigate the relevance of possible confounding variables such as maternal age, ethnicity, and stage of puberty. Details for other possible confounders such as maternal smoking during pregnancy and socioeconomic status were not available. The cohort was further separated into quintiles based on birth weight and the above parameters analyzed by analysis of variance using Fisher's PLSD post hoc test for significance of differences between quintiles. Statistical analyses were performed using GraphPad PRISM software (GraphPad Software, Inc., La Jolla, CA).

## 3. Results

### 3.1. Study Cohort

 The study cohort consisted of 95 subjects (43 male) aged between 10 and 16 years with a mean age of 13.5 ± 0.2 years and selected as having an age-adjusted BMI >95th percentile, a fasting plasma glucose of ≤6 mmol/L, and an A1C of ≤6% (≤7 mmol/L). The mean BMI was 33.0 ± 0.6 and the mean BMI *z-*score was 2.22 ± 0.04 (Table 1). Stage of puberty was assessed for the 69 subjects within the REACH trial, of which 35 were in Tanner stage 5, 7 in stage 4, 14 in stage 3, and 7 in stage 2. Six subjects had not yet entered puberty (stage 1). In the second cohort of 26 subjects all were assessed as being in puberty by the same clinical investigator as for the REACH trial, Cheril Clarson. The mean age, BMI *z-*score, and birth weight did not significantly differ between the two subgroups.

### 3.2. Metabolic and Cardiovascular Risk Factors

 Subject characteristics are depicted in Table 1. The mean HOMA value was slightly elevated at 4.08 ± 0.26 indicating the presence of insulin resistance, with 35% of subjects having a HOMA value greater than 4.0. The mean serum HDL cholesterol level was low at 1.16 ± 0.03 nmol/L, placing this between the 10th and 25th percentiles of age and sex-adjusted values [26], and the mean triglyceride level of 1.19 ± 0.07 nmol/L was elevated between the 90th and 95th percentiles. The HDL cholesterol level was below 1.3 nmol/L in 81% of subjects, whilst 16% had a triglyceride value above 1.7 nmol/L. The mean blood pressure *z-*scores were 0.30 ± 0.08 for systolic and 0.33 ± 0.10 for diastolic, and they indicated only a slight elevation above normal. Only 2 subjects had a blood pressure greater than 130/85 mm. These characteristics did not significantly differ with gender.

Linear regression analysis showed significant positive relationships between BMI *z-*score and the percent body fat, serum triglyceride levels, leptin levels and the leptin/adiponectin ratio, HOMA, and fasting plasma insulin (Table 2). A significant negative relationship was found between BMI *z-*score and serum HDL cholesterol levels, whilst no significant relationship existed with serum adiponectin levels, LDL cholesterol, fasting or 2 h GTT glucose values, or A1C levels. The BMI *z-*score was positively related to blood pressure but not to endothelial function as measured by PAT (Table 2). However, HOMA values showed a significant positive relationship with both fasting and 2 h GTT glucose values. These relationships did not differ with sex or Tanner pubertal stage. Thus, adolescent obesity was associated with insulin resistance, dyslipidemia, and higher blood pressure.

### 3.3. Associations of Birth Weight and Risk Factors

The mean birth weight of the study group was 3527 ± 64 g (males 3487 ± 94 g, females 3560 ± 87 g), the mean length of gestation was 39.9 ± 0.1 weeks, and the mean maternal age was 28.8 ± 0.9 years. Linear regression analysis showed a significant positive relationship between birth weight and BMI *z-*score, suggesting that infants of larger birth weight tended to have the greatest BMI within this population of obese adolescents (Table 3). Stepwise regression showed that this relationship remained significant after accounting for the possible confounding variables of maternal age and stage of puberty. No relationship was found between birth weight and percent body fat. A positive relationship existed between birth weight and serum adiponectin levels, but not with leptin or circulating lipids. However, the leptin/adiponectin ratio was negatively associated with birth weight (Table 3). Fasting plasma insulin levels were negatively associated with birth weight, as were the HOMA values, but no significant relationship existed between birth weight and fasting or 2 h GTT glucose values. A significant positive relationship did exist between birth weight and A1C values. No relationships were found between birth weight and blood pressure *z-*scores or PAT values. Also, birth weight showed no relationship with ethnicity or maternal age, or with the age of menarche, the Tanner pubertal stage, or the presence of acne, hirsutism, or acanthosis in the obese adolescents; the latter two represent indicators of hyperinsulinemia. No significant differences in the slopes of the regression lines were found between males and females for any of the above measurements. The results suggest that obese adolescents of higher birth weight tended to have a higher BMI, but there was no evidence of dyslipidemia. While glycemic control following an OGTT was not compromised, a higher birth weight was associated with a greater A1C, suggesting a greater tendency towards hyperglycemia. In contrast, obese adolescents who were of lower birth weight had a greater incidence of insulin resistance and higher fasting insulin levels. However there was no evidence of a relationship between birth weight and cardiovascular risk factors.

**Figure 1:**
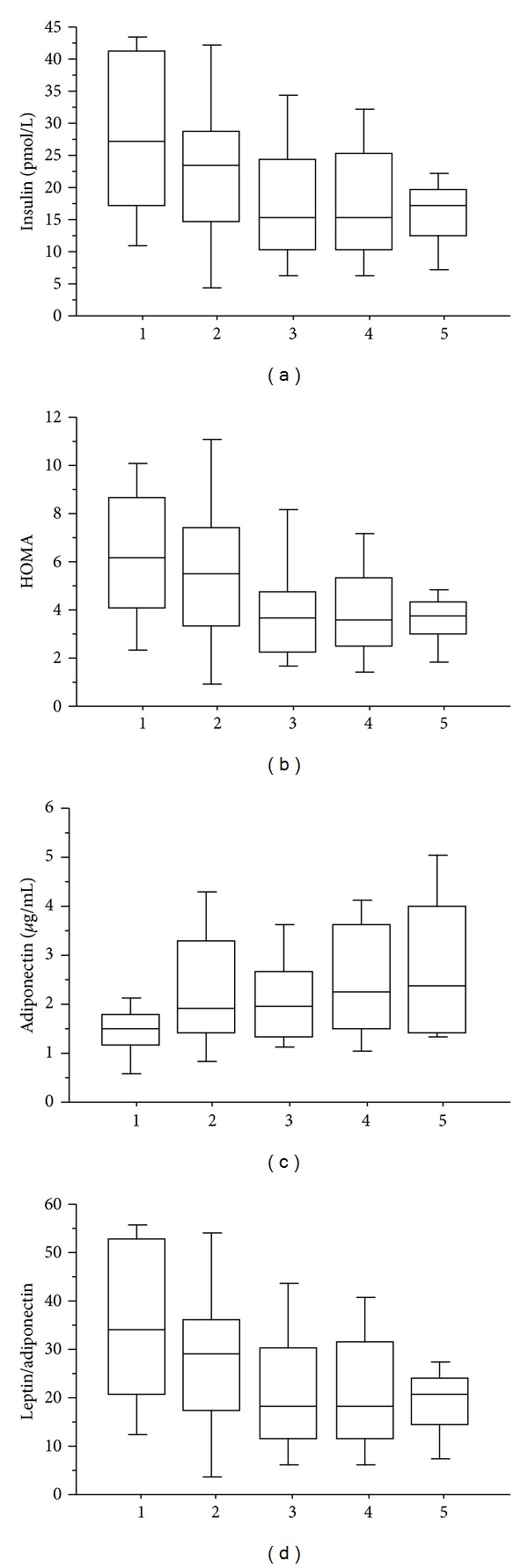
Box plot analysis showing median values and interquartile range for plasma insulin (a), HOMA (b), adiponectin (c), and the leptin/adiponectin ratio (d) in obese adolescents when separated by quintiles representing the range of birth weights for the same individuals. 1: <2500 g [7]; 2: 2500–3000 g [8]; 3: 3001–3500 g [27]; 4: 3501–4000 g [28]; 5: >4000 g [19]. *P* < 0.05 or less for insulin (1 versus 3, 4, and 5), HOMA (1 versus 5), adiponectin (1 and 3 versus 5), and the leptin/adiponectin ratio (1 versus 4 and 5).

To confirm these findings, the birth weights of the study subjects were divided into quintiles, ranging from infants who were small for gestational age (SGA) (<2500 g, *n* = 7) to those who were large for gestational age (LGA) (>4000 g, *n* = 19). Obese adolescents were significantly more likely to have higher fasting insulin levels and greater insulin resistance (HOMA) if they were SGA (Figure 1). Conversely, obese adolescents who were LGA were significantly more likely to have a higher plasma adiponectin levels and a lower leptin/adiponectin ratio. Similarly, LGA infants were significantly more likely to have higher circulating triglyceride levels (not shown). No relationships were found between blood pressure or PAT values and birth size quintile.

## 4. Discussion

As expected, adolescents with a BMI >95th centile (mean BMI *z-*score 2.22) demonstrated a positive correlation between BMI and percent body fat, circulating triglycerides, and leptin. Thirty-five percent of subjects had an HOMA value >4.0, indicating insulin resistance [22]. The HOMA value positively correlated with BMI, but no relationships existed between BMI and fasting glucose values, or the 2 h value from the GTT. Insulin resistance usually increases during puberty, commencing at Tanner stage 2 and peaking at stage 3 for both genders [28]. In our study, no relationship was found between HOMA and Tanner stage, although the distribution of subjects was biased towards stage 5 indicating transition through puberty. Blood pressure *z-*scores were only slightly elevated in the obese subjects. We previously demonstrated a significant inverse relationship between PAT ratio and BMI *z-*score in a cohort of adolescents that included both nonobese and obese individuals, indicating the association of endothelial dysfunction with obesity [25]. In the present study the mean PAT ratio was 1.77, which is lower than that reported by us for nonobese subjects of the same mean age, at 2.06 [25], and indicating that endothelial dysfunction was likely to be present. Thus, obesity in this group of adolescents was associated with the appearance of insulin resistance and evidence of relative vascular dysfunction compared to nonobese adolescents.

In a population of over 1,000 9-year-olds not selected by weight the prevalence of SGA was 7% and LGA 9% [29]. In our smaller cohort of obese subjects, the relative prevalence was biased in favour of LGA (SGA 7% versus LGA 20%). As would be expected, therefore, birth weight at term was positively related to BMI *z-*score in obese adolescents but was not related to percent body fat or circulating leptin levels. Similarly, there were no differences in mean percent body fat or leptin when birth size was arranged by quintiles. Birth weight has a positive correlation with both lean body mass and percent body fat at age 9 or 10 [30]. However, SGA babies can show disproportionately higher fat mass as adults, despite having a lower BMI [20]. Postnatal growth trajectory appears to be at least as important as birth weight in determining the risk of obesity in adolescents or adults [31, 32]. We did not have access to postnatal growth trajectory, but our data show that within a group of obese adolescents, the BMI *z-*score is related, in part, to birth weight, but not percent body fat which may be determined more by postnatal growth patterns.

We noted a strong inverse relationship between birth weight and both HOMA and fasting circulating insulin levels, indicating a relatively greater insulin resistance in obese adolescents who were of smaller birth size. Although fasting glucose and glucose tolerance were not statistically different, glycemic status was worse in these individuals because A1C levels were also significantly higher for those of smaller birth weight. Adolescents who were born SGA had, on average, a HOMA value 2.5 units greater than those born LGA. There is strong evidence that infants born to mothers who were diabetic have increased risks of obesity and T2D as young adults [33], but these infants tend to be LGA and macrosomic. In a nondiabetic *in utero* environment, SGA infants whose mothers were subjected to nutritional restriction as in the Dutch hunger famine of 1944 demonstrated a statistically greater risk of becoming obese as young adults and had a higher rate of T2D [34]. When 34 overweight children were evaluated by birth weight, the HOMA value and measures of tissue oxidative stress were significantly higher for those in the bottom tertile for birth weight compared to the top [21]. Similarly, Norris et al. [27] showed an inverse relationship between birth weight and adult fasting glucose levels, HOMA, and the risk of T2D after adjusting for weight circumference.

We found a strong inverse relationship between circulating adiponectin levels and the adiponectin/leptin ratio, with birth size. Adiponectin increases hepatic triglyceride accumulation, enhances pancreatic beta cell function, and improves insulin sensitivity in peripheral tissues [35]. A decreased release of adiponectin from adipocytes is associated with obesity, and circulating levels correlate with insulin resistance and are inversely related to the risk of developing T2D [36]. It is therefore likely that the greater insulin resistance observed in obese adolescents who were smaller at birth may be mechanistically linked to a lower availability of adiponectin. The altered circulating adiponectin may reflect an altered location of adipose stores depending on whether the subjects were smaller or larger at birth since circulating levels decrease with increasing truncal obesity [37].

While this cohort of obese adolescents had relatively decreased mean PAT ratios and slightly elevated mean blood pressure compared to a nonobese population, these measurements showed no relation to birth weight. Several studies found that birth size was inversely related to systolic and diastolic blood pressure in children or adults and to risk of cardiovascular disease [38, 39]. However, Filler et al. [40] found that blood pressure *z-*scores in childhood did not correlate with birth weight but were related to maternal prepregnancy BMI. Our findings suggest that for obese adolescents, birth size does not confer a detectable risk of future cardiovascular problems.

In summary, this study shows that the individual BMI, but not the percent body fat, in obese adolescents was positively related to birth weight. While no differential risk for development of cardiovascular disease was found that could be related to birth weight, there was significantly worse glycemic status and insulin resistance in obese adolescents of lower birth weight. This represents an additional risk factor for future T2D in obese adolescents and may be relevant to the intensity of lifestyle or pharmaceutical intervention necessary within an obesity prevention program.

## Figures and Tables

**Table 1:** Subject characteristics (mean ± S.E.M, [range]).

Age (years)	13.5 ± 0.2 (9.8–16.9)
Birth weight (g)	3527 ± 64 (1899–4990)
BMI	33.0 ± 0.6 (21.8–46.1)
BMI *z*-score	2.22 ± 0.04 (1.18–3.00)
Systolic BP *z*-score	0.30 ± 0.08 (−1.71–3.24)
Diastolic BP *z*-score	0.33 ± 0.10 (−2.41–3.09)
PAT (RH-PAT)	1.77 ± 0.04 (1.00–2.73)
FPG (Mmol/L)	4.93 ± 0.05 (3.00–6.00)
2 h GTT glucose (mmol/L)	5.59 ± 0.15 (2.80–9.80)
Insulin (pmol/L)	18.3 ± 1.11 (2.0–51.0)
HOMA	4.08 ± 0.26 (0.35–12.69)
HDL cholesterol (mmol/L)	1.16 ± 0.03 (0.62–2.57)
LDL cholesterol (mmol/L)	2.36 ± 0.07 (0.69–4.35)
Triglycerides (mmol/L)	1.19 ± 0.07 (0.30–4.20)
Leptin (ng/mL)	38.8 ± 1.75 (9.32–86.20)
Adiponectin (*μ*g/mL)	2.49 ± 0.13 (0.68–7.20)
Leptin/adiponectin ratio	19.6 ± 1.2 (3.0–53.0)
A1C (%)	5.4 ± 0.1 (4.7–6.0)
Body fat (percent)	45.5 ± 0.1 (35.5–56.3)

Values show median and range. BMI: body mass index; BP: blood pressure, PAT: peripheral arterial tomography; HOMA: Homeostasis Model Assessment. A normal distribution of values for each variable was confirmed using the Kolgomorov-Smirnov test. Values were obtained from 95 individuals, except for values for A1C and percent body fat, which were obtained from a sub-group of 69 subjects. The mean age, BMI *z*-score, and birth weight of this sub-group did not significantly differ from the larger cohort.

**Table 2:** Relationships between BMI *z*-score or HOMA and metabolic and vascular parameters in obese adolescents.

	*r* ^2^	*P* value	Slope-intercept
Regression analysis versus BMI *z*-score			
Body fat (%)	0.44	<0.001	*y* = 0.27 + 0.08*x*
Systolic BP *z*-score	0.07	0.007	*y* = −0.84 + 1.04*x*
Diastolic BP *z*-score	0.08	0.005	*y* = 0.31 + 0.74*x*
Insulin	0.24	<0.001	*y* = −13.25 + 14.22*x*
HOMA	0.21	<0.001	*y* = −2.86 + 3.12*x*
HDL cholesterol	0.05	0.026	*y* = 1.60 − 0.20*x*
Triglycerides	0.05	0.027	*y* = 0.24 + 0.43*x*
Leptin	0.18	<0.001	*y* = −4.98 + 19.74*x*
Leptin/adiponectin ratio	0.05	0.030	*y* = 3.50 + 7.22*x*
Regression analysis versus HOMA			
FPG	0.16	<0.001	*y* = −6.89 + 2.22*x*
2 h GTT glucose	0.14	0.002	*y* = −0.06 + 0.62*x*

BMI: body mass index; BP: blood pressure; HOMA: Homeostasis Model Assessment; GTT: glucose tolerance test. No significant relationships were found between BMI *z*-score and adiponectin, LDL, fasting blood glucose or 2 h GTT values, and A1C or peripheral arterial tomography.

**Table 3:** Relationships between birth size (g) and metabolic and vascular parameters in obese adolescents.

	*r* ^2^	*P* value	Slope-intercept
BMI *z*-score	0.05	0.033	*y* = 0.77 + 1.28*E* − 4*x*
Insulin	0.05	<0.001	*y* = 32.39 − 0.01*x*
HOMA	0.05	<0.001	*y* = 7.42 − 0.01*x*
A1C	0.08	0.020	*y* = 0.05 + 0.26*E* − 6*x*
Adiponectin	0.07	0.008	*y* = 0.55 + 0.01*x*
Leptin/adiponectin ratio	0.07	0.010	*y* = 37.20 − 0.01*x*

BMI: body mass index; HOMA: Homeostasis Model Assessment. No significant relationships were found between birth size and BMI, percent body fat, leptin, basal glucose or 2 h GTT values, systolic or diastolic blood pressure *z*-scores, peripheral arterial tomography, HDL, LDL, or triglyceride values, maternal age, ethnicity, age of menarche, Tanner puberty stage, or the presence of acne, hirsuitism, or acanthosis.
